# Neurologic outcomes for adult spinal cord ependymomas stratified by tumor location: a retrospective cohort study and 2-year outlook

**DOI:** 10.1007/s10143-023-02166-2

**Published:** 2023-09-29

**Authors:** Keanu Chee, Grégoire P. Chatain, Michael W. Kortz, Stephanie Serva, Keshari Shrestha, Timothy H. Ung, Jens-Peter Witt, Michael Finn

**Affiliations:** 1 Department of Neurosurgery, University of Colorado Anschutz Medical Campus School of Medicine, 12605 E 16th Ave, Aurora, CO 80045 USA; 2 Department of Neurosurgery, Thomas Jefferson University Sidney Kimmel Medical College, Philadelphia, PA USA

**Keywords:** Ependymoma, McCormick Neurologic Scale, Spine, Tumor location

## Abstract

Determine whether craniocaudal spinal cord tumor location affects long-term neurologic outcomes in adults diagnosed with spinal ependymomas (SE). A retrospective cohort analysis of patients aged ≥ 18 years who underwent surgical resection for SE over a ten-year period was conducted. Tumor location was classified as cervical, thoracic, or lumbar/conus. Primary endpoints were post-operative McCormick Neurologic Scale (MNS) scores at < 3 days, 6 weeks, 1 year, and 2 years. One-way ANOVA was performed to detect significant differences in MNS scores between tumor locations. Twenty-eight patients were identified. The average age was 44.2 ± 15.4 years. Sixteen were male, and 13 were female. There were 10 cervical-predominant SEs, 13 thoracic-predominant SEs, and 5 lumbar/conus-predominant SEs. No significant differences were observed in pre-operative MNS scores between tumor locations (*p* = 0.73). One-way ANOVA testing demonstrated statistically significant differences in post-operative MNS scores between tumor locations at < 3 days (*p* = 0.03), 6 weeks (*p* = 0.009), and 1 year (*p* = 0.003); however, no significant difference was observed between post-operative MNS scores at 2 years (*p* = 0.13). The mean MNS score for patients with thoracic SEs were higher at all follow-up time points. Tumors arising in the thoracic SE are associated with worse post-operative neurologic outcomes in comparison to SEs arising in other spinal regions. This is likely multifactorial in etiology, owing to both anatomical differences including spinal cord volume as well as variations in tumor characteristics. No significant differences in 2-year MNS scores were observed, suggesting that patients ultimately recover from neurological insult sustained at the time of surgery.

## Introduction

Ependymomas are benign, non-infiltrative neuroepithelial tumors that arise from the ependymal cells lining the cerebral ventricles and spinal cord [1, 2]. There is a bi-modal age distribution with most ependymomas being diagnosed at either 0–4 years old or 55–59 years old. In adults, ependymomas tend to arise more frequently in the spine, with spinal ependymomas (SE) comprising 45% of all intramedullary spinal cord tumors [2, 3]. According to the World Health Organization (WHO), ependymomas can be classified into three categories: WHO Grade I (myxopapillary and subependymoma), WHO grade II (classic, cellular, papillary, clear cell, and tanycytic), and WHO Grade III (anaplastic) [4]. Generally, WHO Grade I ependymomas are the least aggressive subtypes, while anaplastic ependymomas have the most malignant potential; however, there can be significant variations in tumor location, ease of resection, and potential for tumor recurrence within each ependymoma class [3].

Surgical resection represents the standard of care for SEs with extent of resection consistently being considered the most important factor for determining long-term prognosis [3, 5, 6]. Histologic classification is also an important prognostic factor as WHO Grade I and II SEs have been shown to have improved survival benefits [2, 7, 8]. However, in comparison to histologic grade, some studies have suggested that tumor location may be a more accurate predictor of prognosis [9–11]. A recent study found that SEs located in the lower spine (thoracic, thoracolumbar, and conus + cauda equina) have significantly shorter progression-free survival (PFS), as well as a higher potential for recurrence despite having a lower WHO grade in comparison to SEs arising in the upper spine (cervicomedullary, cervical, and cervicothoracic) [9]. The impact of tumor location on survival outcomes for SE has been reinforced in several studies [2, 12, 13].

Neurologic deficits can also vary significantly based on tumor location [9]. As SEs grow, they can cause progressive myelopathy due to compression of adjacent spinal cord tissue [14]. Following tumor resection, transient neurological deterioration may also be seen in cases where separation of normal tissue from tumor is hampered by an ill-defined tumor capsule [4]. While tumor location has been shown to convey considerable prognostic value in regard to overall survival (OS), PFS, and the rate of recurrence, the association between tumor location and post-operative neurological outcomes remains unclear. Therefore, this study aims to determine whether craniocaudal spinal cord tumor location affects neurologic outcomes in adults who underwent resection of SE. We hypothesize that SEs arising in the cervical spine demonstrate worse neurologic deficits due to the involvement of pathways that involve both the upper and lower extremity.

## Methods

### Patient selection

We conducted a single-center retrospective cohort study to assess whether neurologic outcomes varied by tumor location among patients aged 18 years or older who have undergone surgery for SE. All patients underwent surgery at the University of Colorado Hospital from January 1, 2010 to December 31, 2020. Clinical data were collected using the electronic medical records. This study was reviewed by the Colorado Multiple Institutional Review Board and determined to be exempt from review. Patient consent was not required.

### Evaluated parameters and outcomes

Patients’ age, sex, tumor grade (WHO I–III), pre- and post-operative functional neurologic status, extent of tumor resection, post-operative complications, tumor recurrence rate, and follow-up duration were noted. The functional neurologic status was retrospectively classified according to the McCormick Neurologic Scale (MNS; 1 = neurologically intact; ambulates normally; none-to-minimal dysesthesia, 2 = mild motor/sensory deficit; maintains functional independence, 3 = moderate neurological deficit; limitation of function; independent with external aid, 4 = severe motor or sensory deficit; limited function with dependence on a wheelchair or cane/brace; usually not independent, 5 = para- or quadriplegic) both pre- and post-operatively at < 3 days, 6 weeks, 1 year, and 2 years [14]. Patients were excluded from this study, if they did not have adequate pre- and post-operative MNS scores recorded or lost to follow-up in the post-operative period.

Radiological parameters included the spinal levels of the tumor, as well as tumor location. Spinal level was determined based on observing how many levels the solid tumor spanned. Tumor location was classified into three different regions based on the predominant location along the craniocaudal axis at which the tumor resided (i.e., cervical-predominant, thoracic-predominant, or lumbar/conus-predominant). Tumor location was determined by a board-certified neuro-radiologist and confirmed by the authors. Radiographic evidence of tumor recurrence was also confirmed by a board-certified neuro-radiologist. The primary outcome was assessed by comparing post-operative MNS scores between each tumor location at each post-operative follow-up time.

### Statistical analysis

Data storage and analysis were performed with Prism 9 (GraphPad Software, San Diego, CA, USA). Cohort summary is provided by descriptive statistics and are reported as mean ± standard deviation (*SD*) or as simple proportions and percentages. Independent variables included age, sex, follow-up time, extent of resection (GTR vs STR), and tumor grade (WHO 1–3); Non-parametric data were expressed as means ± *SD* using one-way Kruskal-Wallis analysis of variance (ANOVA) to compare mean MNS for each post-operative follow-up time between tumor locations. A *p*-value < 0.05 was considered statistically significant.

## Results

### Patient demographics and tumor characteristics

Twenty-eight patients were included in our analysis. The mean age was 44.4 ± 15.6 years. Sixteen (57.1%) were male, and 12 (42.9%) were female. For tumor location, 10 (35.7%) SEs were cervical-predominant, 13 (46.4%) were thoracic-predominant, and 5 (17.9%) were lumbar/conus predominant. The mean follow-up times were 55.9 ± 57.1 months in the cervical-predominant cohort, 71.2 ± 44.8 months in the thoracic-predominant cohort, and 36.2 ± 23.1 months in the lumbar/conus-predominant cohort (*p* = 0.136) (Table 1).

**Table 1:** Summary of patient demographics and clinical outcomes

	Patients (*n* = 28)		
Tumor location			
Cervical	*n* = 10 (35.7%)		
Thoracic	*n* = 13 (46.4%)		
Lumbar	*n* = 5 (17.9%)		
Gender (male/female)	16 (57.1%)/12 (42.9%)		
Age (years)	44.4 ± 15.6		
WHO grade (by tumor location)	Cervical	Thoracic	Lumbar
WHO Grade I	*n* = 2	-	*n* = 5
WHO Grade II	*n* = 8	*n* = 12	-
Undetermined	**-**	*n* = 1	-
Extent of resection			
Gross total resection	*n* = 7	*n* = 11	*n* = 5
Sub-total resection	*n* = 3	*n* = 2	-
Evidence of recurrence			
	*n* = 3	*n* = 4	-
Total follow-up time (months)	55.9 ± 57.1	71.2 ± 44.8	36.2 ± 23.1

For tumor grades per location, in the cervical-predominant group, eight (80%) tumors were WHO grade II, while two (20%) were WHO grade I tumors. For thoracic-predominant SEs, 12 tumors (92.3%) were WHO grade II, while 1 (7.7%) tumor had an undetermined grade. For lumbar/conus-predominant SEs, all 5 tumors were WHO grade I (Table 1).

### Neurologic outcomes

**Fig. 1:**
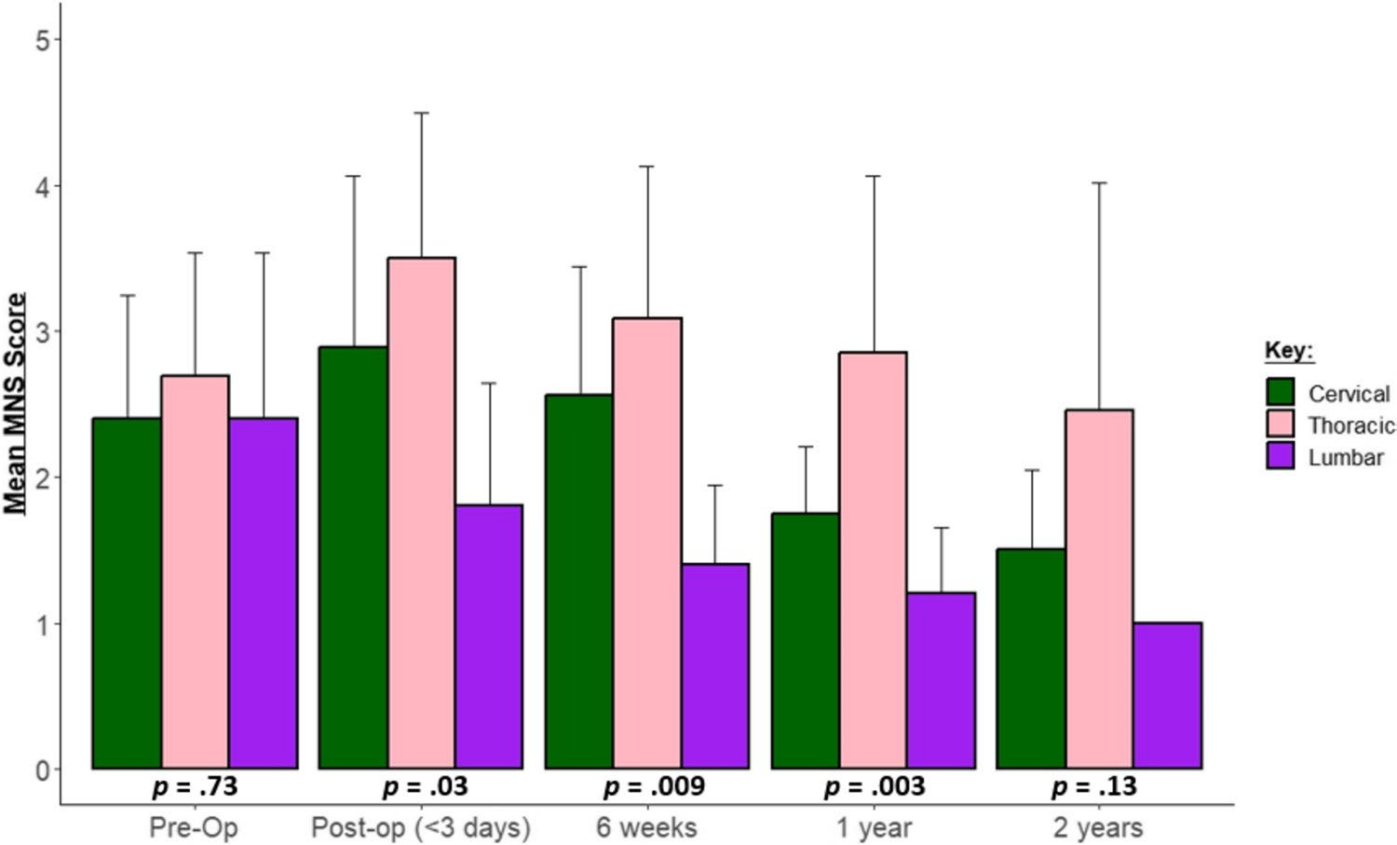
Grouped bar chart showing average postoperative MNS scores at preop, < 3 days, 6 weeks, 1 year, and 2 years. Green depicts patients with cervical-predominant pathology; pink demonstrates patients with thoracic-predominant tumor while purple shows patients suffering from lumbar/conus tumors. Mean preoperative MNS was found to be similar in patients regardless of craniocaudal tumor location for (*p* = 0.73). At < 3 days, 6-week and 1 year post-operative time points, the mean MNS in the cervical, thoracic, and lumbar/conus groups were significantly different (*p* = 0.03, *p* = 0.009, and *p* = 0.003 respectively). No significant difference in MNS scores at the 2-year follow-up time point was observed

Mean pre-operative MNS was found to be similar in patients regardless of craniocaudal tumor location for cervical, thoracic, and lumbar/conus groups (2.40 ± 0.84, 2.69 ± 0.86, and 2.40 ± 1.14, respectively; *p* = 0.73) (Fig. 1).

In the immediate post-operative period (< 3 days), the mean MNS scores in the cervical, thoracic, and lumbar/conus groups were 2.89 ± 1.17, 3.50 ± 1.00, and 1.80 ± 0.84, which was significantly different (*p* = 0.03). At the 6-week post-operative time point, the mean MNS which were also found to be significantly different in the cervical, thoracic, and lumbar/conus groups were 2.56 ± 0.88, 3.08 ± 1.04, and 1.40 ± 0.55, respectively (*p* = 0.009). At the 1-year mark, the mean MNS in the cervical, thoracic, and lumbar/conus groups were 1.75 ± 0.46, 2.85 ± 1.21, and 1.20 ± 0.45 respectively (*p* = 0.003). Lastly, At the 2-year post-operative time point, the mean MNS scores in the cervical, thoracic, and lumbar/conus groups were 1.50 ± 0.55, 2.45 ± 1.57, and 1.0 ± 0.00, respectively; however, the differences between means did not achieve statistical significance (*p* = 0.13) (Fig. 1).

**Fig. 2:**
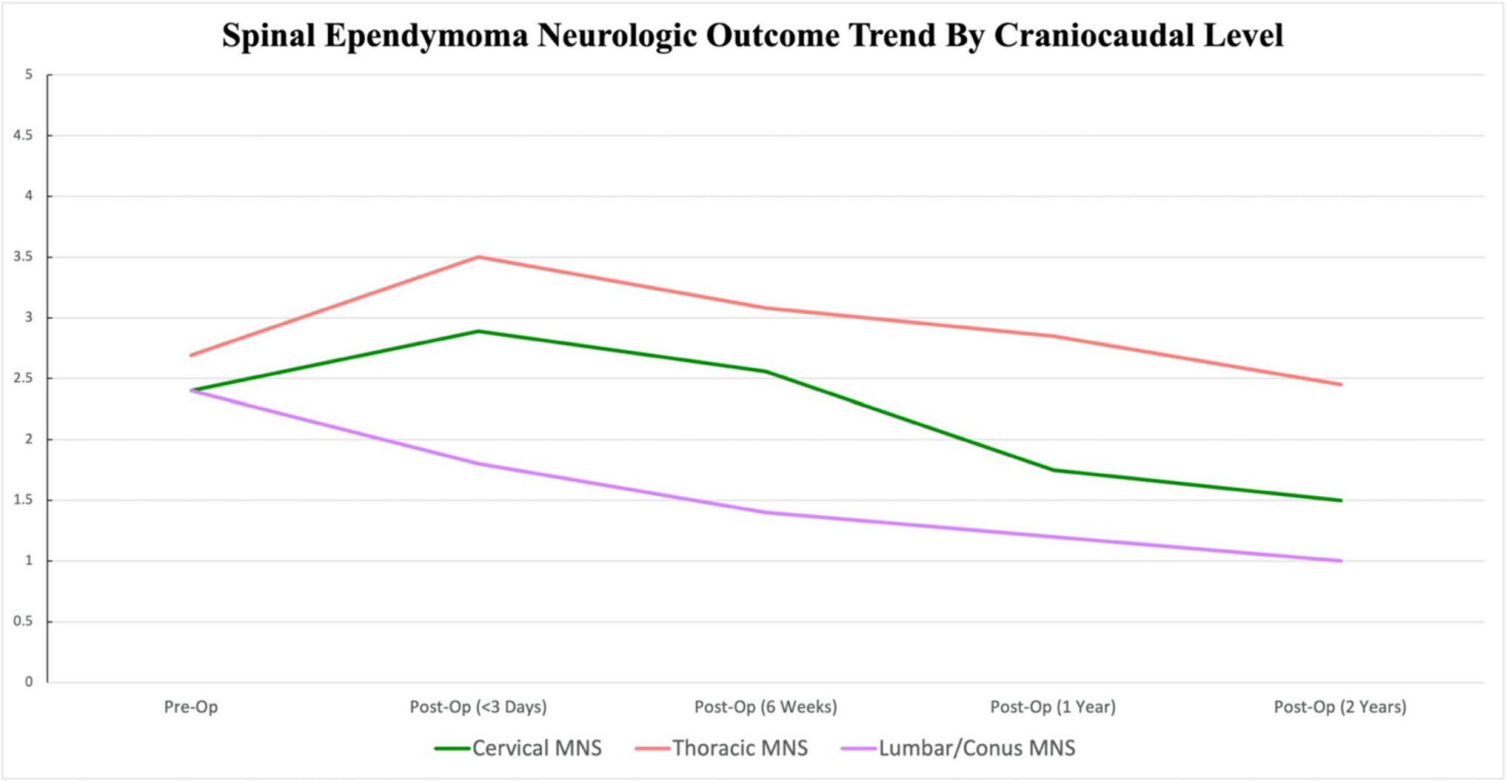
Overall trendline for MNS scores between SE groups. The overall trendline for MNS scores preoperatively to 2 years postoperatively demonstrated higher MNS scores in the thoracic group compared to other craniocaudal spinal levels

The overall trendline for MNS scores pre-operatively to 2 years post-operatively demonstrated higher MNS scores in the thoracic group compared to other craniocaudal spinal levels (Fig. 2).

### Post-operative complication

Three (10.7%) patients with thoracic-predominant SEs experienced worsened unilateral or bilateral lower extremity weakness post-operatively; however, all patients had considerable improvement in their symptoms at the time of discharge. One (3.6%) patient with a thoracic-predominant SE developed new post-operative atrial fibrillation managed conservatively. One (3.6%) patient with a cervical predominant SE developed a post-operative urinary tract infection; however, no wound complications were noted in our cohort. No patients with a lumbar/conus-predominant SE experience post-operative complications. No patients required a return to the operating room within 30 days of their initial admission.

### Extent of resection and tumor recurrence rates

Gross total resection was achieved in 7 (70%) patients with a cervical-predominant SE, 11 (84.6%) patients with a thoracic-predominant SE, and in 5 (100%) patients with a lumbar/conus-predominant SE.

Radiographic evidence of tumor recurrence was noted in 3 (10.7%) patients with a cervical-predominant SE and 4 (14.3%) patients with a thoracic-predominant SE. For recurrent cervical-predominant SEs, one patient underwent surgery for further tumor resection followed by adjuvant radiotherapy, while two patients were referred for radiotherapy without surgery. For recurrent thoracic-predominant SEs, one patient underwent surgery for further tumor resection followed by adjuvant radiotherapy, while one patient only underwent surgery for further tumor debulking. Repeat surgery was attempted in one patient with a thoracic-predominant recurrent tumor, though was stopped due to loss of motor evoked potential during resection. One patient with a thoracic-predominant recurrent tumor was referred for repeat surgery, though was unfortunately lost to follow-up. A summary of demographic and clinical data is presented in (Table 1).

## Discussion

Spinal ependymomas are benign neuroepithelial tumors that primarily occur in adults [2, 3]. Several studies have studied the effects of tumor location on survival outcomes, noting that SEs located in the lower spinal cord regions had a tendency to recur earlier and more frequently in comparison to the SEs arising in the upper spinal cord regions [9, 10]. Such outcomes may be related to lower spinal cord SEs being more histologically aggressive with greater tumor invasion into healthy adjacent spinal cord tissue leading to both lower rates of gross total resection, and an increased potential for tumor recurrence [10]; the reasons for these observations remain unclear. Even though tumor location has been shown to impact patients’ survival outcomes, no studies have investigated whether there is a concomitant association between tumor location and neurologic outcomes. To the authors’ knowledge, this is the first study to ascertain whether craniocaudal spinal cord tumor location is associated with post-operative neurologic outcomes in adults diagnosed with SE.

Despite our hypothesis that neurologic outcomes would be worse for SEs arising in the cervical spinal cord, our study instead demonstrated that patients diagnosed with SEs in the thoracic spinal cord suffered worse post-operative neurologic outcomes in comparison to SEs arising elsewhere out to 1 year. This is corroborated by the observation that patients with thoracic SEs were consistently shown to have higher MNS scores at all post-operative time points, although was insignificantly different at the 2-year mark. This result is somewhat surprising given that lesions affecting the cervical cord compromise somatomotor pathways for both the upper and lower extremities, which would result in greater neurological dysfunction [15]. Nonetheless, there does appear to be an important relationship between tumor location and post-operative neurological outcomes. Interestingly, we also observed that patients who were found to have multilobulated cystic-intra-tumoral components on their pre-operative magnetic resonance imaging had worse post-operative neurologic outcomes than those without a cystic tumoral component; however, no statistically significant difference was observed when stratifying by tumor location. Additionally, we also observed that tumor size, as determined by the number of consecutive levels that the tumor spans, did not significantly correlate with worse post-operative neurologic outcomes.

Several factors may explain the relationship of the worse neurological outcomes of thoracic SEs compared to SEs arising in other regions of the spinal cord. The extent of resection has been considered the most important factor for determining long-term survival in patients with SE [12]. For any tumor, the goal is safe maximal resection. For SEs, this is highly dependent on whether there is a well-defined tumor capsule, which may vary by WHO grade. Tumors with distinct capsules have correlated to fewer post-operative neurologic deficits, likely due to easier distinction between tumor and healthy tissue, which would then facilitate safer resection and greater preservation of healthy tissue [4, 16, 17]. In comparison to WHO grade I and II SEs, WHO grade III SEs (anaplastic) have a higher tendency to invade surrounding tissue and could lower the potential for safe gross total resection leading to worse post-operative neurologic deficits [4, 18]. However, in our cohort, all patients had either a WHO grade I or II SE, and all thoracic-predominant tumors were classified as WHO grade II. Therefore, it is less likely that thoracic SE tumor grades and subsequent extent of tumor resection would sufficiently explain why patients with thoracic SEs have worse post-operative neurologic outcomes. Furthermore, other studies have already shown that extent of tumor resection does not correlate with post-operative neurologic function [12, 19].

Instead, it is more likely that intrinsic tumor characteristics, as well as anatomical differences throughout the spine have greater influence on post-operative neurologic outcomes in patients with thoracic SEs. Anatomically, the thoracic spinal cord and corresponding canal have the lowest diameter along the spinal axis [20]. Therefore, SEs arising in the thoracic spine may ultimately confer more compressive damage to a greater ratio of healthy cord tissue resulting in worse post-operative neurologic deficits. It has also been proposed that a limited blood supply to the thoracic cord, in conjunction with prolonged tumor compression, may also increase the vulnerability to iatrogenic cord damage, particularly during surgical manipulation [4, 21].

Our study also consistently showed that patients with lumbar/conus-predominant SEs had the lowest post-operative MNS scores at all follow-up time points. Although the lumbar spinal cord has a smaller diameter than the cervical spinal cord, it transitions to the cauda equina; therefore, SEs that grow in the lumbar region have a more neurologically forgiving space [20, 22]. As a result, SEs arising in the lumbar region may not produce the same magnitude of mass effect and compressive burden on the spinal cord compared to SEs that arise in the cervical or thoracic cord, resulting in less neurologic deficits in these patients.

Pre-operative functional status has also been shown to be an important prognostic factor for determining post-operative neurological outcomes [12]. Despite the lack of significant difference among pre-operative MNS scores, patients with thoracic SEs had the highest pre-operative MNS score. As such, it is a rational observation that these patients also have the highest corresponding post-operative MNS scores in comparison to cervical and lumbar/conus SEs. However, although the thoracic SE cohort maintained the highest post-operative MNS scores through 2 years of follow-up, no significant differences between post-operative MNS scores were observed between all groups at the 2-year follow-up time point. This finding might suggest that at the 2-year follow-up time point, patients with thoracic SEs have had a long enough time to recover from their initial neurologic insult. Furthermore, our patients’ neurosurgical care is often supplemented with rigorous physical therapy to maximize functional neurologic recovery throughout the post-operative period, which may also contribute to the lack of significant differences in MNS scores at the 2-year follow-up time point.

## Limitations

This study is not without its limitations. Given the retrospective design of our study and low sample size, this may introduce sampling bias. Also, our small cohort of patients treated at a single center impacts the generalizability of our results. Specifically, lumbar ependymomas generally represent the more common location for spinal ependymomas; however, lumbar ependymomas have the lowest representation in our study cohort, thereby decreasing the external validity of these results.

Second, although MNS scores are a validated measure of assessing patients’ functional status, the assessment requires a subjective investigator evaluation, which could introduce confirmation, measurement, or historical bias. Third, while we did not observe a significant correlation between tumor size and post-operative neurologic outcomes, our study may have been underpowered to detect a difference. Lastly, we did not characterize patients’ post-operative rehabilitation regimen or follow-up care, both of which have significant impacts on neurologic recovery. Given the findings of this work, future prospective studies should aim to assess various factors that may impact functional neurological outcomes in patients with SEs.

## Conclusion

Tumor location is an important factor in determining post-operative functional neurologic outcomes in adult patients diagnosed with SE. Tumors arising in the thoracic SE are associated with worse neurologic deficits in comparison to SEs arising in other spinal regions. This is likely multifactorial in etiology, owing to both anatomical differences throughout the spinal cord as well as variations in tumor characteristics; however, the precise etiology for this observation remains unclear. Further research is necessary to determine the factors that contribute to thoracic SEs being associated with worse neurologic outcomes.

## Data Availability

Not applicable
